# Research on Vault Settlement during Three-Step Tunnel Construction Process Based on Sandstone Rheological Experiment

**DOI:** 10.3390/ma17184619

**Published:** 2024-09-20

**Authors:** Chang Peng, Yong Qu, Helin Fu, Chengda Xie, Guiqian Cao

**Affiliations:** 1 China Construction Fifth Engineering Bureau Civil Engineering Co., Ltd., Changsha 410011, China; 18274818664@163.com (C.P.); 17775756436@163.com (Y.Q.); 2 School of Civil Engineering, Central South University, Changsha 410075, China; 234812211@csu.edu.cn (C.X.); guiqiancao@csu.edu.cn (G.C.)

**Keywords:** granite, sandstone, rheological properties, experimental study, Burgus model, Cvisc model

## Abstract

Tunnel stability is influenced by the rheological properties of the surrounding rock. This study, based on the Ganshen high-speed railway tunnel project, examines the rheological characteristics of siltstone and sandstone through laboratory tests and theoretical analysis. Rheological curves and parameters are derived, revealing the time-dependent deformation mechanisms of the surrounding rocks. A numerical simulation model is created using these parameters to analyze deformation and stress characteristics based on different rock levels and inverted arch closure distances. Results indicate that sandstone follows the Cvisc model, with the Maxwell elastic modulus increasing under higher loads while the viscous coefficient decreases. The vault displacement is mainly affected by the surrounding rock strength; lower strength leads to greater displacement, which also increases with the closure distance of the inverted arch. These findings are crucial for determining the optimal closure distance of inverted arches in sandstone conditions.

## 1. Introduction

The inverted arch is a reverse arch-shaped structure set at the bottom of the tunnel to improve the stress conditions of the upper support structure. It is one of the main components of the tunnel structure. The timely closure of the inverted arch is an important step in the construction of the New Austrian Tunneling Method. According to current specifications, the closure position of the inverted arch in rock masses of grades III, IV, and V, which are classified by the RMR method, should not exceed 35 m from the excavation face, and strict requirements are imposed on the excavation advance. However, with the introduction of large-scale mechanized equipment, the efficiency of tunnel operations has greatly improved, resulting in less time required for the same advance. This leads to the closure of the inverted arch in tunnels before the deformation of the surrounding rock is fully completed, causing cracks in the secondary lining. In response to this issue, China Railway Group has abolished document No. 120, which specifies the distance for inverted arch closure; however, the highway department still enforces the current specifications, leading to limitations and inadequacies in guiding mechanized construction operations and an inability to meet the requirements of tunnel construction progress and the layout step distance of mechanized equipment. In order to fully exert mechanized construction efficiency under the premise of ensuring construction safety and quality, it is urgently necessary to carry out research on the rheological properties of surrounding rocks for excavation advance and closure of the inverted arch.

Regarding the study of the rheological properties of surrounding rocks, many scholars have conducted relevant research. Lu Kaijing [1] divided the surrounding rock into a plastic flow zone, a plastic softening zone, a plastic hardening zone, and an elastic zone based on consideration of the rheological properties of the surrounding rocks. Wang Tao [2], by combining the Burgers model with an elastic-plastic model, studied the deformation properties of accelerated creep stages in the rock deformation process. Yao Yangping [3] established a three-dimensional elastic-viscous-plastic constitutive model to study the deformation rheology of soil-like surrounding rocks. Guo Shuntao [4], based on experiments and combining theory and numerical simulation, studied the creep deformation of muddy sandstone under high stress. Wang Gang [5] considered the anchor rod as a one-dimensional Kelvin model and the surrounding rock as a three-dimensional Burgers model, revealing the coupled rheological mechanism between the anchor rod and the surrounding rock. Yao Jun [6] conducted triaxial compression tests to investigate the rheological properties of fine sandstone and coarse sandstone. However, there is limited research on the rheological properties of the same rock in different surrounding rock types and stress conditions in practical tunnel engineering.

While the aforementioned studies have significantly contributed to the understanding of the rheological properties of surrounding rocks, there are several critical areas that warrant further examination. For instance, Lu Kaijing’s [1] classification provides a foundational framework, but it may oversimplify the complex transitional behaviors between different zones. The categorization into plastic flow, softening, hardening, and elastic zones assumes distinct boundaries, which in reality could be more fluid and interdependent. This raises questions about the applicability of such a model in heterogeneous rock masses found in situ. Wang Tao’s [2] integration of the Burgers model with an elastic-plastic model is innovative, yet it primarily addresses accelerated creep stages without sufficiently exploring the initial and steady-state creep behaviors, which are equally crucial for comprehensive tunnel design. Similarly, Yao Yangping’s [3] three-dimensional elastic-viscous-plastic constitutive model, while advanced, may not fully capture the anisotropic nature of soil-like rocks, which can exhibit direction-dependent properties that significantly influence their rheological behavior. Guo Shuntao’s [4] combination of experimental data with numerical simulation is commendable, but the reliance on laboratory-scale experiments raises concerns about the scalability of the findings to real-world conditions. Laboratory conditions often fail to replicate the exact stress, moisture, and temperature conditions present in deep tunnel environments, potentially leading to discrepancies in predicted versus actual behavior. Wang Gang’s [5] coupled rheological model of anchor rods and surrounding rock is a step forward in understanding the interaction between support structures and rock mass. However, the assumption of a one-dimensional Kelvin model for the anchor rod might be an oversimplification, considering the complex three-dimensional stress distributions and potential failure modes in practical applications. Yao Jun’s [6] triaxial compression tests provide valuable insights into the rheological properties of different sandstones, yet the study does not account for the long-term effects of environmental factors such as water ingress, chemical weathering, and freeze-thaw cycles, which can significantly alter the mechanical properties of rocks over time.

In fragile and fractured surrounding rock, the inverted arch structure is commonly utilized to bolster the stiffness of tunnel support, enhancing the mechanical performance and controlling overall deformation. This plays a crucial role in the tunnel support structure. Scholars have investigated the mechanism of the inverted arch, with Wang et al. [7] effectively halting the extreme deformation of loess tunnels using a combination of temporary steel arch and temporary inverted arch. Shreedharan et al. [8] discovered that the inverted arch tunnel could be more effective in reducing roof sag and floor heave under existing geo-mining conditions. Sung et al. [9] disclosed the mechanical behavior characteristics of tunnel construction in weak rock using finite difference software and established a tunnel support system.

The use of inverted arches in tunnel support is a well-established practice, yet the current research predominantly focuses on specific case studies or isolated conditions. Wang’s study on loess tunnels, for example, demonstrates the efficacy of temporary supports, but it does not address the long-term stability and potential maintenance issues that could arise. Shreedharan’s findings on roof sag and floor heave reduction are promising, but the study’s reliance on specific geo-mining conditions limits its generalizability to other geological settings. Sung’s use of finite difference software provides a robust analytical approach, but the model’s assumptions and boundary conditions must be critically examined to ensure they accurately reflect the complexities of real-world tunnel environments.

In tunnels excavated by the bench cut method, the invert closure distance greatly influences the deformation and pressure of the surrounding rock. Ye, Wu et al. [10] explored the surrounding rock pressure and supporting internal force variation characteristics of the tunnel excavated by the three benches and seven steps to reserve core soil method. Chen et al. [11] discovered that in tunnels excavated by the “three benches” method, early closure time of the inverted arch can significantly enhance the stability of the surrounding rock and diminish construction risks.

The bench-cut method’s impact on surrounding rock deformation and pressure is a critical area of study, yet the existing research often overlooks the dynamic nature of construction processes and their influence on rock behavior. Ye, Wu’s investigation into pressure and internal force variations provides valuable data, but it may not fully capture the transient effects of excavation and support installation. Chen’s emphasis on early closure time is insightful, yet the practical challenges of implementing such measures in complex tunnel projects need further exploration.

Designing tunnel support in weak rock masses is a time-intensive task for tunnel engineers [12]. If the design is flawed, reshaping works become mandatory. To increase its accuracy, extensive data collection is needed during the preliminary period. The support design is then created using this data. Numerical modeling is the preferred method today [13]. The more the field conditions are reflected in the model, the more accurate the results. Optimum design helps prevent loss of time and money [14]. Aksoy C.O. et al. [15] employed numerical analysis to simulate the mechanical behavior of the tunnel rock mass more precisely.

The reliance on numerical modeling for tunnel support design in weak rock masses is a double-edged sword. While it offers the potential for high precision and optimization, the accuracy of these models is heavily dependent on the quality and comprehensiveness of the input data. Aksoy C.O.‘s work on numerical analysis underscores the importance of detailed field data, yet acquiring such data can be logistically challenging and costly. Furthermore, numerical models often involve simplifications and assumptions that may not fully capture the inherent variability and unpredictability of geological conditions. This necessitates a cautious approach, combining numerical results with empirical observations and iterative adjustments during the construction phase to ensure the reliability and safety of tunnel support systems.

In conclusion, while significant progress has been made in understanding and modeling the rheological properties of surrounding rocks and the design of tunnel support systems, there remain critical gaps and challenges. Future research should aim to address these limitations by incorporating more comprehensive field data, exploring the long-term effects of environmental factors, and developing more sophisticated models that better reflect the complex behaviors of rock masses and support structures in diverse geological settings.

However, under varying surrounding rock conditions, the invert closure distance also displays significant differences in different construction methods. Therefore, the invert closure distance during tunnel excavation should be adapted to local conditions [16]. Moreover, the surrounding rock exhibits time-deformation characteristics. When rheological properties are taken into account, the stability analysis of the tunnel becomes particularly complex. This presents new requirements for the distance between the excavation face and the invert closure. There are few studies on the spatial influence of rheological tunnels on excavation steps and invert closure distance [17]. Based on this, we rely on the Songshan Tunnel and Shimen Gang Tunnel projects of the Jiangxi-Shenzhen High-speed Railway under construction to study the impact of tunnel elevation arch excavation on tunnel deformation under different surrounding rock conditions. This is done using numerical analysis software, with the aim to further enhance the relevant specifications and technical requirements and provide basic research on large-scale mechanized operation [18].

Therefore, this paper conducts graded incremental pressure tests on mudstone and siltstone in laboratory conditions to study their rheological properties. By analyzing the experimental data, rheological models compatible with their deformation properties are constructed [19]. With the use of experimental data, reference values for model-related parameters under different surrounding rock conditions and stress conditions are provided, along with comparative analysis, to reveal the deformation mechanism of the time effect of surrounding rocks [20]. The numerical simulation model with the rheological parameters of the tunnel in siltstone and sandstone excavated by the three-step method is established, revealing the deformation and stress characteristics by different surrounding rock levels and inverted arch closure distance.

## 2. Test Equipment and Specimen Preparation

### 2.1. Test Equipment

**Figure 1:**
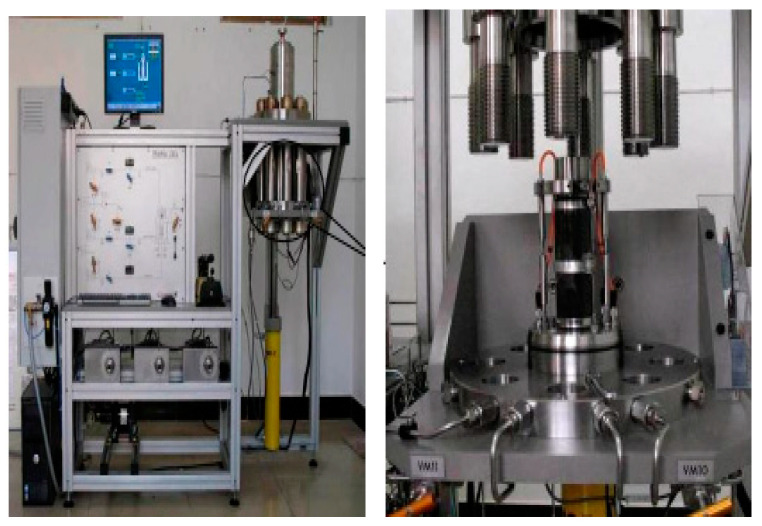
Rock fully automatic triaxial compression servo.

The fully automatic rock triaxial compression servo machine used in the experiment, as shown in Figure 1, is imported from France. This equipment is capable of full computer control and analysis, with fully automated operation, ensuring safe, real-time, and accurate analysis of the entire creep process and achieving digital mapping of the entire creep process.

### 2.2. Specimen Preparation

The silty sandstone used in the experiment was taken from the surrounding rock of the Ganshen high-speed railway tunnel project. The dry blocks were cut and polished to prepare cylindrical specimens. According to the recommended standards of the International Society for Rock Mechanics (ISRM), the drilled rock core specimens need to be prepared with a size of 50 mm × 100 mm (diameter × length). The specimens used in the test were collected from the same location to ensure the same rock properties. There were a total of three groups of sandstone triaxial creep test specimens, with three specimens in each group. Totally 9 specimens. The surrounding rock grades of the specimens were grade III, grade IV, and grade V. However, there were some errors in the actual operation process, so the average size of specimens was indicated in Table 1.

To ensure that the results of the experiment are not influenced by specimen factors, strict selection was carried out on the processed specimens. Firstly, specimens with obvious damage and visible cracks on the surface were removed, and then specimens whose dimensions and smoothness did not meet the requirements were eliminated. The specimens were numbered for convenient processing of experimental results.

The related physical and mechanical parameters are tested first, as shown In Table 2, in which *ρ* is the density, *E_S_* is the elastic modulus, *υ* is Poisson’s ratio, *c* is the cohesion, *φ* is the internal friction angle, and *σ_c_* is the uniaxial compressive strength.

## 3. Rheological Test of Silty Sandstone

### 3.1. Test Schemes

The experiment was conducted using a single-step incremental loading method. The rock specimens were subjected to a triaxial creep test and the conventional triaxial compression test. The conventional triaxial test helps to understand the basic mechanical properties of the rock and the ultimate load under corresponding conditions, which is useful for determining the load magnitude during the creep test. For the triaxial creep test, considering the complex geological environment and stress levels of the tunnel, a multi-stage load creep testing approach is adopted.

During the experiment, the confining pressure was set to 10 MPa, and the stress loading path was the same for each group of rock specimens. The stress loading conditions for the sandstone specimens are shown in Table 3. The testing machine automatically collected the experimental data, with a data acquisition interval of 0.1 h. The loading schemes were as follows:

(1) The confining pressure was provided to the target value at a rate of 0.2 MPa/s.

(2) Apply the axial loading for the rock specimens at a rate of 2.55 MPa/min until it reaches the first axial stress level. The duration of this stress level was set to 2 h.

(3) The subsequent axial loading process was applied in the same manner as step two until the rock failure occurred.

**Figure 2:**
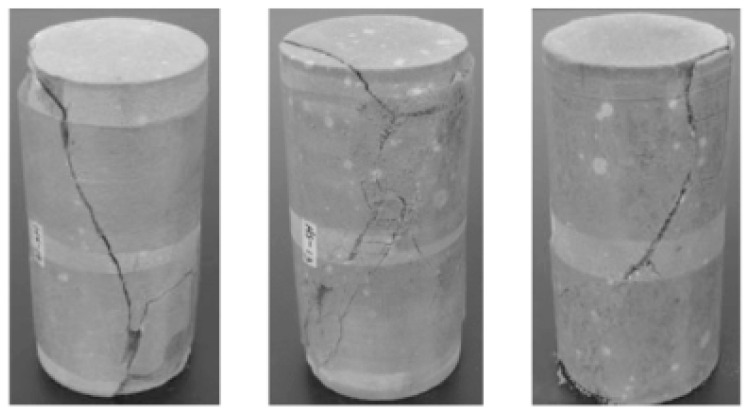
Failure Image.

The failure condition of the sandstone specimens is shown in Figure 2.

### 3.2. Test Results

Considering the discreteness of rock properties, each group of stones in the experiment was tested at least three times. The median level of the three specimens was selected as the reference for the entire experiment, and corresponding test curves were plotted.

**Figure 3:**
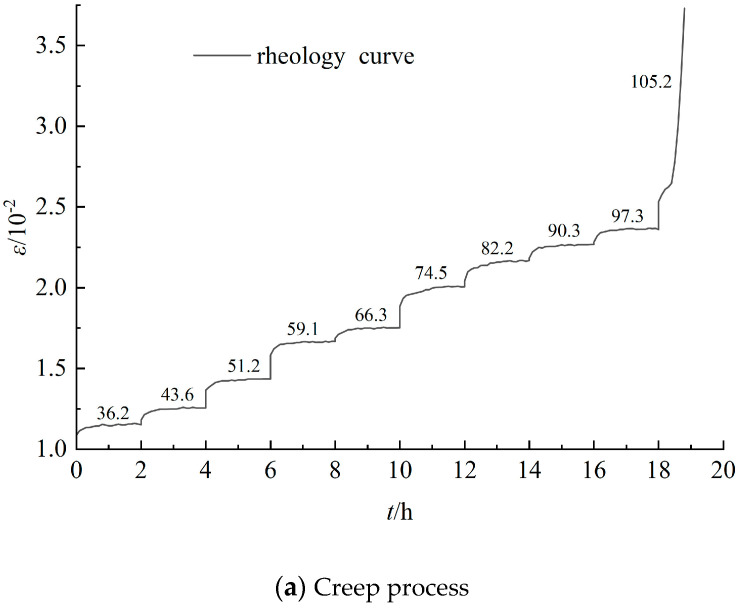

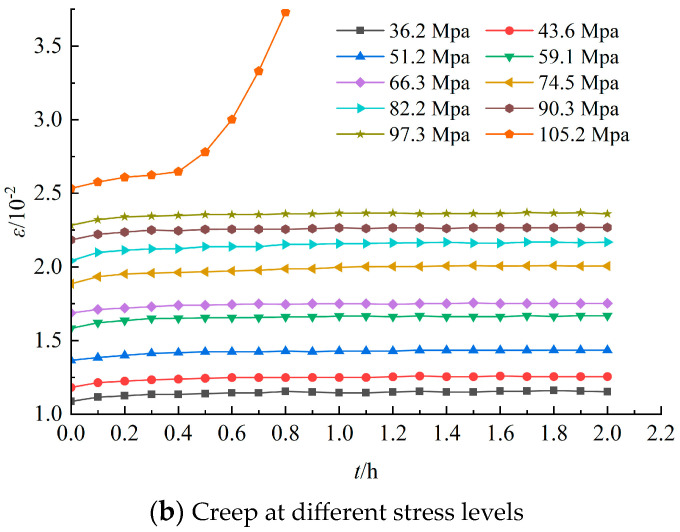
Rheological curves of grade III sandstone surrounding rocks under various loads.

**Figure 4:**
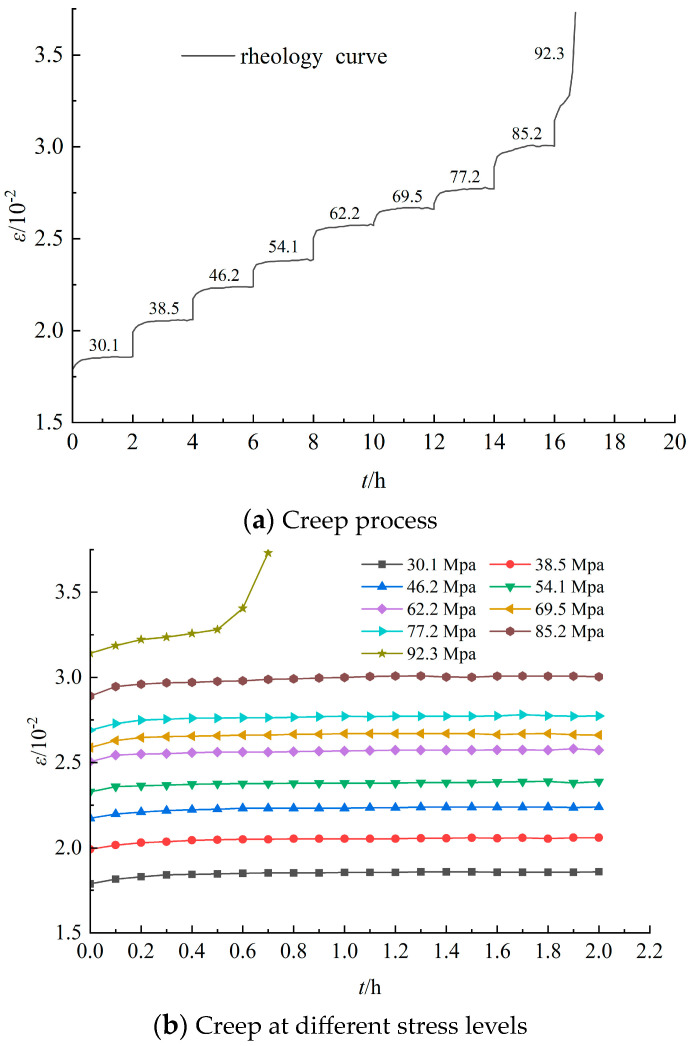
Rheological curves of grade IV sandstone surrounding rocks under various loads.

**Figure 5:**
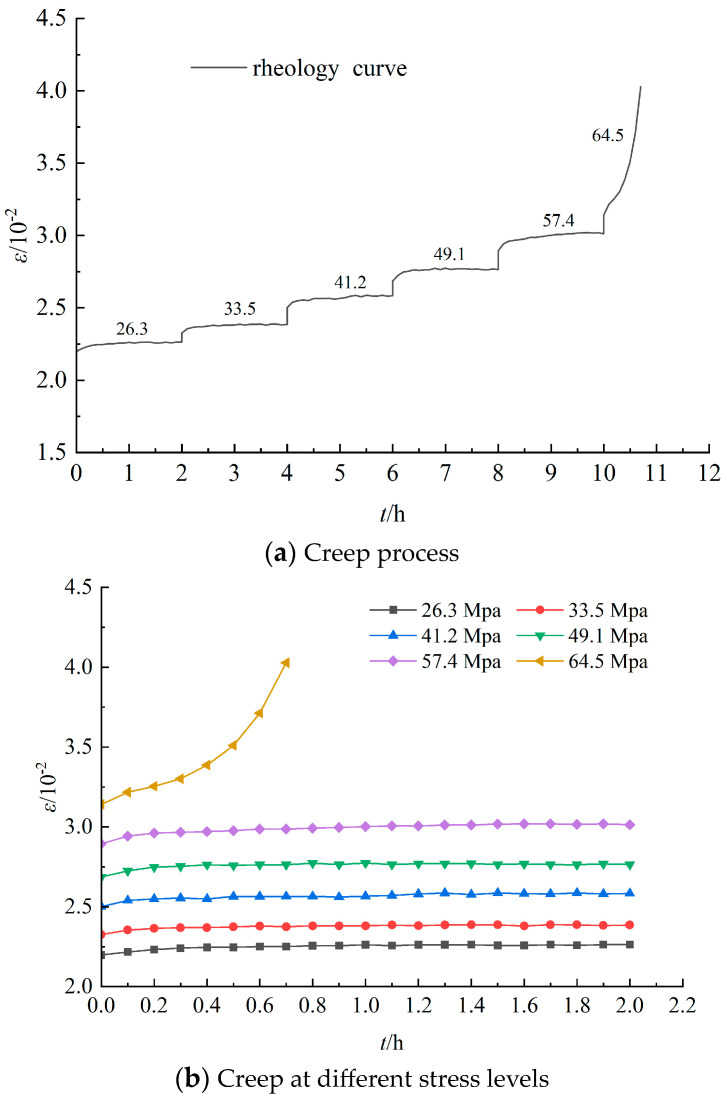
Rheological curves of grade V sandstone surrounding rocks under various loads.

Analyzing the data from the sandstone creep test, Figure 3, Figure 4 and Figure 5 were obtained, representing the complete creep curve for grade III, grade IV, and grade V surrounding rock, as well as the creep curves at different stress levels. The creep of rocks generally manifests in three traditional phases, namely instant elastic strain, primary creep, secondary creep, and tertiary creep [21,22,23,24,25,26,27]. The instant elastic strain is reversible and can take place promptly when a constant load is applied. Primary creep is ephemeral and usually exhibits a decelerated strain rate. Secondary creep is the steady phase, whereas tertiary creep is the final phase, which is generally characterized by an accelerated strain rate until the creep life is exhausted. From Figure 3, Figure 4 and Figure 5, it can be observed that the compressive strength of the sandstone is significantly lower than that of the siltstone. Moreover, when the load approaches its ultimate strength, significant instantaneous plastic deformation occurs on the basis of creep until failure. Additionally, under the same stress level, the elastic-plastic instantaneous deformations increase with the increase in the surrounding rock grade. The post-instantaneous deformation creep curves have steeper slopes as the surrounding rock grade and stress level increase, indicating more pronounced rheological properties.

### 3.3. Cvisc Model for Rheological Testing of Silty Sandstone

**Figure 6:**
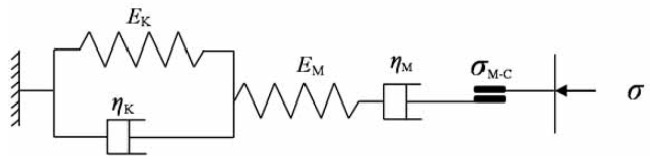
Cvisc rheological model.

In the calculation and analysis process of tunnel deformation, the selection of the constitutive model for the tunnel is crucial. Therefore, it is necessary to find an appropriate constitutive model to describe the creep characteristics of the surrounding rock. Based on the above test results, analysis reveals the following three properties of the creep curves of the sandstone: (1) The early creep stage can be represented by the Maxwell rheological model; (2) the later creep stage can be represented by the Kelvin rheological model; (3) before the failure of the specimen, the siltstone exhibits significant plastic deformation, suggesting the presence of a frictional element connected in series with the model [28,29]. Summarizing the properties of the creep curves of the rock specimens, it is found that the Cvisc creep model aligns well with the experimental data. The Cvisc rheological model is a viscoelastic-plastic rheological model composed of the Burgers model and a plastic element in series, as shown in Figure 6 [30,31].

In the figure, *E_M_* and *η_M_* are the elastic modulus and viscosity coefficient of Maxwell model respectively. *E_K_* and *η_K_* are the elastic modulus and viscosity coefficient of the Kelvin model respectively.

The corresponding rheological equation for the Cvisc model is as follows:

(1) $$\varepsilon (t)=\frac{\sigma_{0}}{E_{M}}+\frac{\sigma_{0}}{\eta_{M}}t+\frac{\sigma_{0}}{E_{K}}\left(1-e^{-\frac{E_{K}}{\eta_{K}}}\right)+\varepsilon_{p}$$

In Equation (1), *E_M_* and *E_K_* are the elastic modules of Maxwell body and Kelvin, respectively; *η*_M_ and *η*_K_ are the viscosity coefficients of Maxwell and Kelvin, respectively; *ε_p_* is the plastic strain.

### 3.4. Determination and Analysis of Cvisc Model Parameters

For the parameter calculation in the model construction, using the nonlinear least squares fitting function, the regression inversion of model parameters in Equation (1) is performed to obtain the Cvisc model parameters for each surrounding rock grade. The Origin data analysis software is user-friendly, allowing most data analysis tasks to be completed simply by selecting commands from the menu, which facilitates data fitting. Moreover, Origin includes a large number of built-in functions, and it also supports user-defined mathematical functions to meet additional research needs.

Based on the above principles, to make fitting with Origin more convenient, the equation is as follows:

*K*_1_ = *σ*_0_/*E_M_*, *K*_2_ = *σ*_0_/*η*_M_, *K*_3_ = *σ*_0_/*E_K_*, *K*_4_ = *E_K_*/*η*_K_, *K*_5_ = *ε_p_*, *ε*(*t*) = *y*, *t* = *x* (2)

So, the Equation (1) transforms to the following:

(3) $$y=K_{1}+K_{2}+K_{3}x-K_{2}e^{-K_{4}x}+K_{5}$$

**Figure 7:**
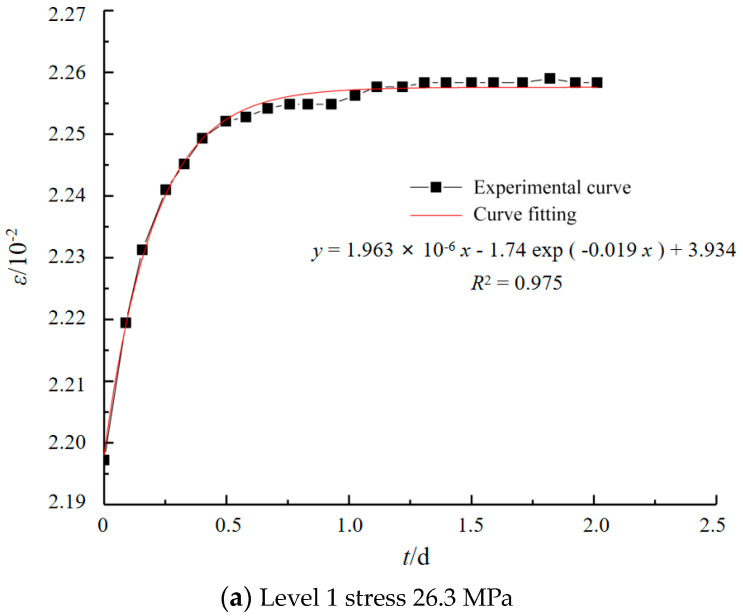

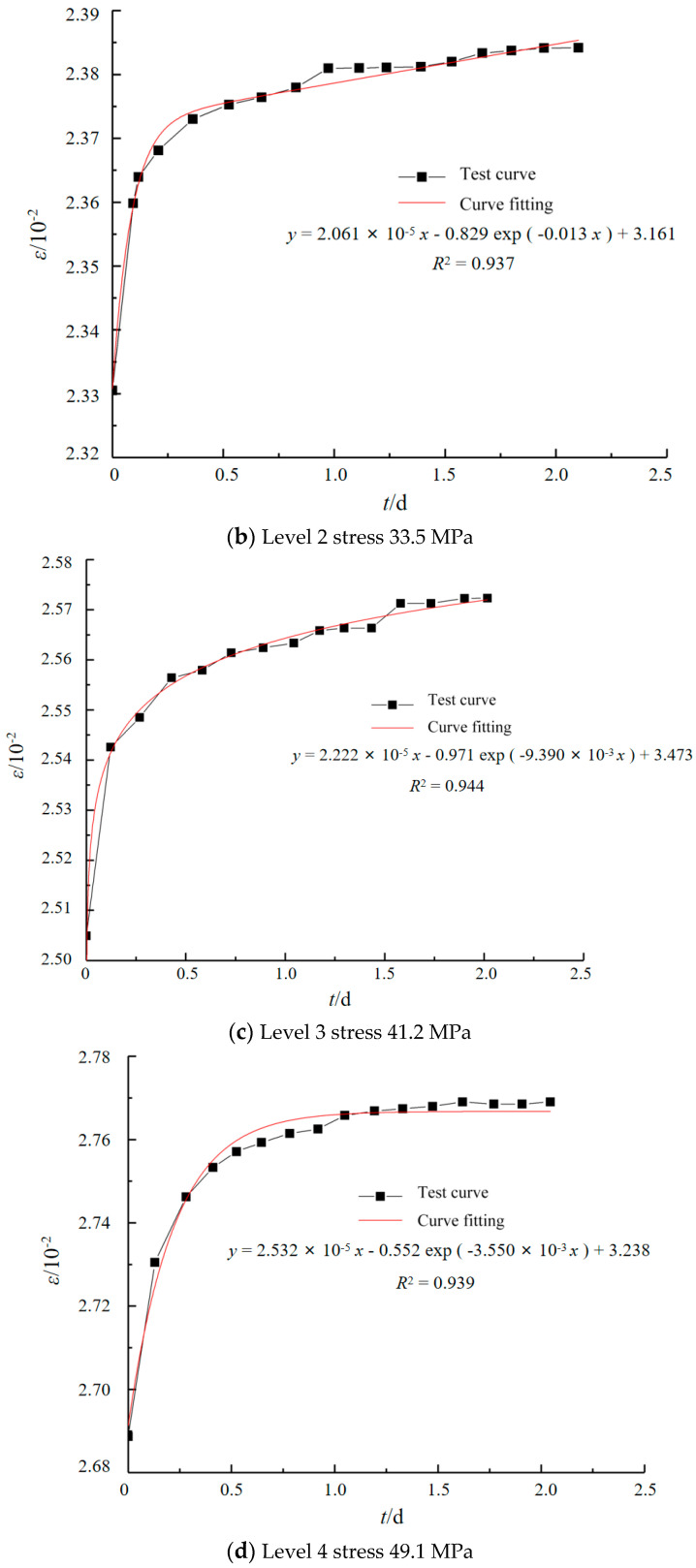

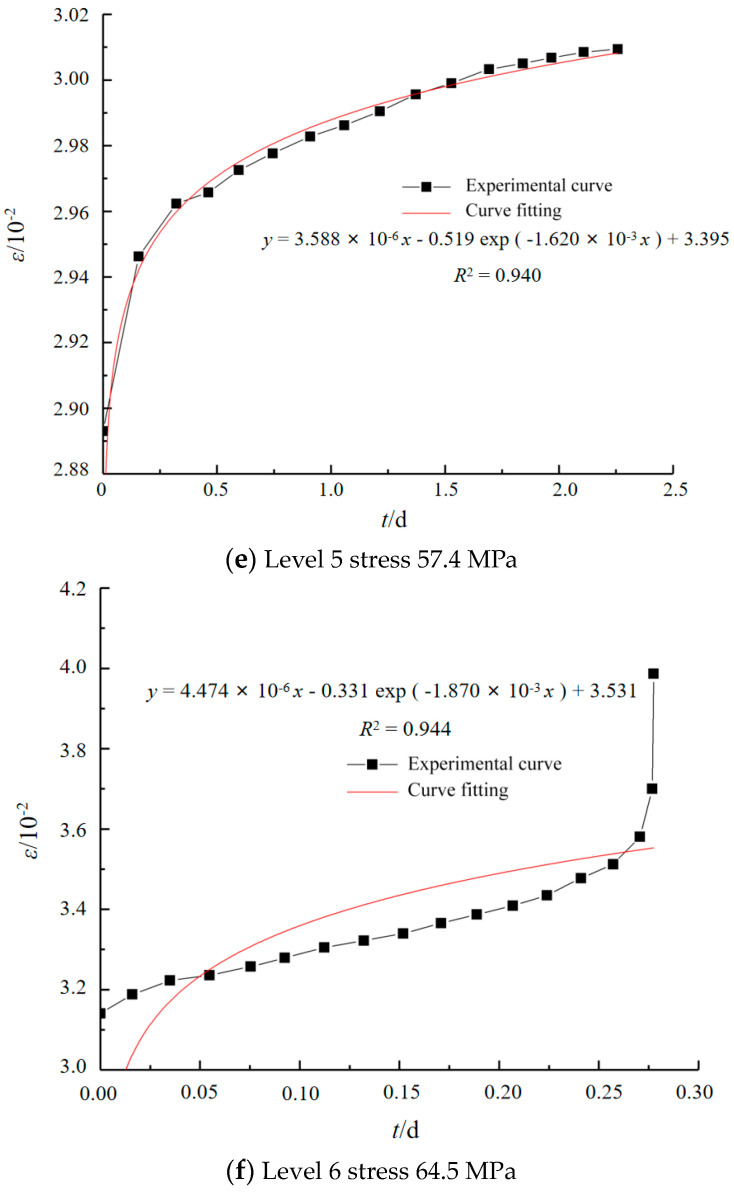
Surrounding rock test and Cvisc rheological model.

Using Origin’s custom fitting function feature, based on the Nonlinear Least Squares Fitting method, the model is tuned to ensure that the sum of the squared differences (errors) between the predicted values and the actual observed values is minimized during the fitting process. Taking grade V siltstone as an example, the comparison between the experimental curve and the fitted curve is shown in Figure 7.

**Figure 8:**
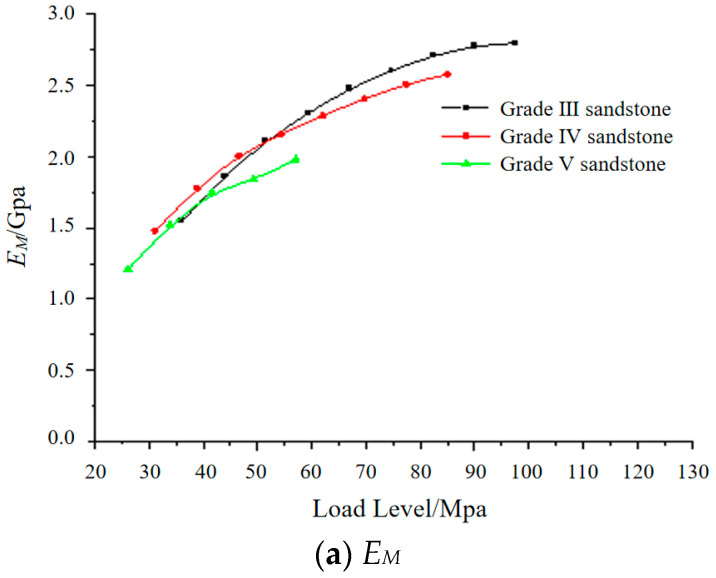

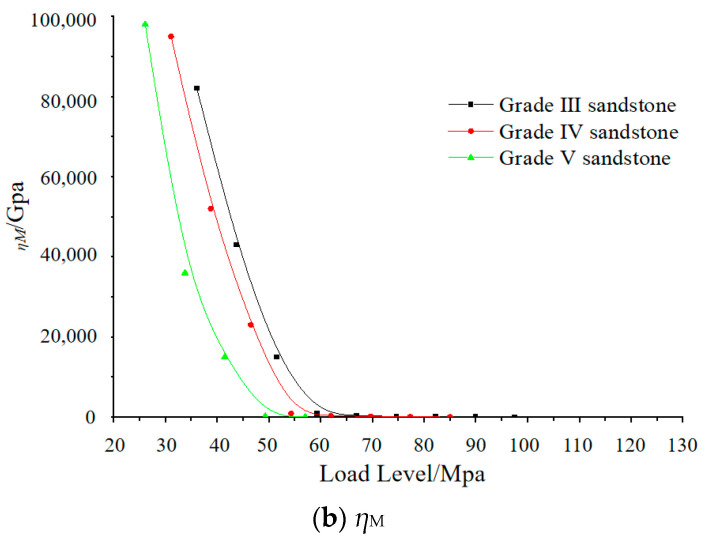
Maxwell model parameter change diagram.

**Figure 9:**
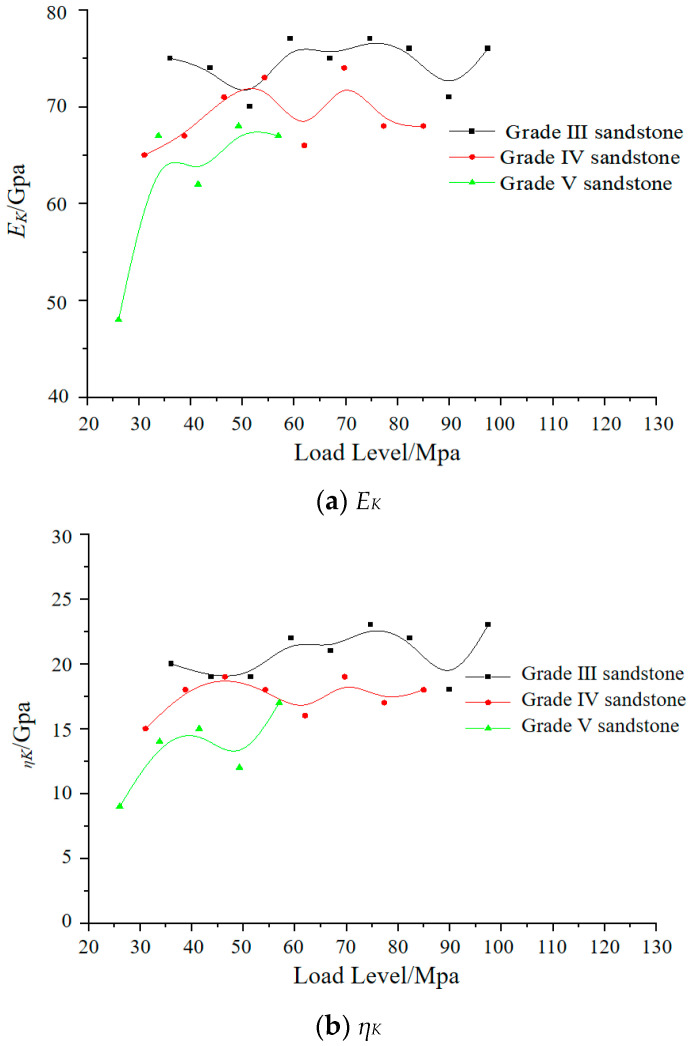
Kelvin model parameter change diagram.

The recommended parameters for each level of surrounding rock are obtained by fitting the Cvisc model, and the changes of each parameter *E_M_*, *η*_M_, *E_K_,* and *η*_K_ are plotted under different stress levels and surrounding rock levels, as shown in Figure 8 and Figure 9.

From Figure 8 and Figure 9, it can be observed that as the surrounding rock grade decreases and the rock conditions improve, both the elastic modulus and viscosity coefficient of the Cvisc rheological model increase to some extent. However, the differences between the parameters of different surrounding rock grades are relatively small.

Analyzing the creep mechanical parameters, it is found that the Maxwell elastic modulus increases with the increase in axial stress level, but the rate of increase gradually decreases. This is because sandstone undergoes a relatively significant consolidation phase during the initial loading stage, causing an increase in the elastic modulus. The Maxwell viscosity coefficient reflects the creep rate during the constant strain rate creep stage. The smaller the value, the higher the creep rate during the steady-state creep stage. As the axial stress level increases, the creep rate also increases, while the fitted creep mechanical parameter generally decreases with the increase in axial stress level, which matches the actual creep behavior of the rock. However, the variations of the Kelvin creep parameters and in the Cvisc model are not obvious, but they fluctuate around a certain level. The above experimental research has laid the foundation for the selection and application of the constitutive model in the numerical simulation process.

## 4. Numerical Modeling Calculation

### 4.1. Numerical Model Establishment

**Figure 10:**
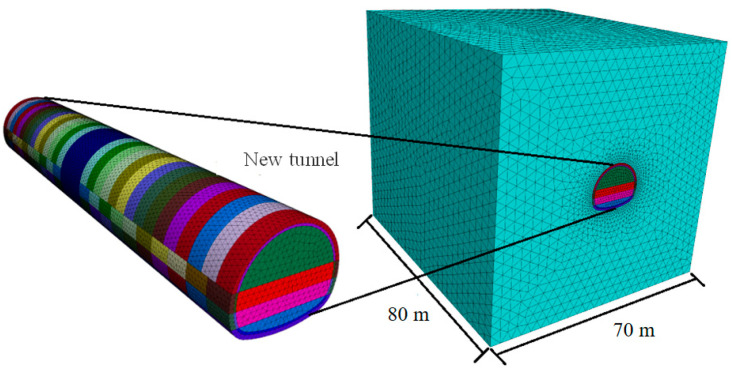
Numerical analysis and calculation model.

By utilizing FLAC3D6.0 software in conjunction with Rhyceros 5 3D external modeling software, we constructed a numerical excavation model to examine the impact of tunnel excavation on the surrounding rock mass, the excavation technique, and the properties of the support system. The model was designed following the tunnel construction steps depicted in Figure 10, featuring dimensions of 80 m along the tunnel’s longitudinal Y-axis and 70 m crosswise to the tunnel, a burial depth of 20 m, and a lower section depth of 30 m. The top boundary of the model was set as a free boundary condition, whereas the remaining sides and the bottom were assigned normal restraint boundary conditions. The stress boundary condition was defined by the self-weight stress field.

The initial support and temporary support adopt a solid unit, the second liner adopts a Shell unit, and the anchor adopts the Cable unit provided by the software. The total number of nodes is 207,800, and the total number of units is 149,700.

The boundary conditions are set as follows: the four sides of the model are set as normal displacement constraints, the bottom surface of the model is set as complete constraints, and the upper surface of the model is free.

The grid sensitivity is set as follows: if the numerical model mesh size is too large, the calculation accuracy will be affected, while if the mesh size is too small, the calculation cost will be increased. Therefore, in this paper, a hexahedral mesh with a soil mesh size of 1 m is selected, which is suitable for the creep effect analysis of tunnel excavation.

We have considered the most unfavorable situation of the relying tunnel, which is the construction process of the V-grade surrounding rock, and simulated it as built by three-step method construction [32,33]. To examine the effect of varying excavation steps on tunnel deformation, the excavation steps of 30 m, 40 m, 45 m, 50 m, 55 m, and 60 m are taken as calculation conditions. To examine the effect of the initial excavation length of the inverted arch on tunnel deformation, the initial excavation lengths of the inverted arch of 3 m, 4 m, 5 m, and 6 m are taken as calculation conditions.

### 4.2. Field Monitoring

As the article mentioned above, different excavation steps and inverted arches are applied to site construction.

By monitoring the vertical displacement and horizontal convergence at the top of the tunnel, information such as deformation and stability of the tunnel’s surrounding rock can be obtained, and the rationality of construction methods, support types, and related parameters can be evaluated, so as to improve data support for the analysis of the temporal and spatial effects of tunnel construction and deformation control technology and ensure the smooth progress of construction. During tunnel construction, a multi-point displacement meter is used to monitor the settlement at the top of the tunnel section and the horizontal convergence of the arch waist. Three sets of instruments are deployed. The monitoring values of tunnel vault settlement are shown in Table 4.

**Figure 11:**
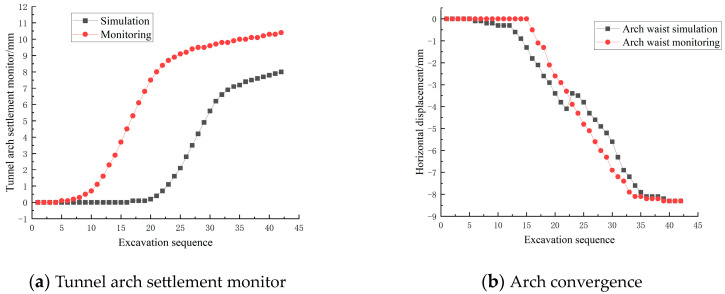
Comparison of simulated values and monitored values.

The comparison between the simulated values of the model and the monitored values is shown in Figure 11. The monitoring values of the left and right arches are 8.0 mm and 10.4 mm, respectively, and the monitoring values of the left and right arches of the leading caves are 10.2 mm and 7.6 mm, respectively. It can be seen that the error between the simulated values of the model and the monitored values is about 7%, and the changing trend is basically the same.

### 4.3. The Step of the Tunnel Excavation

**Figure 12:**
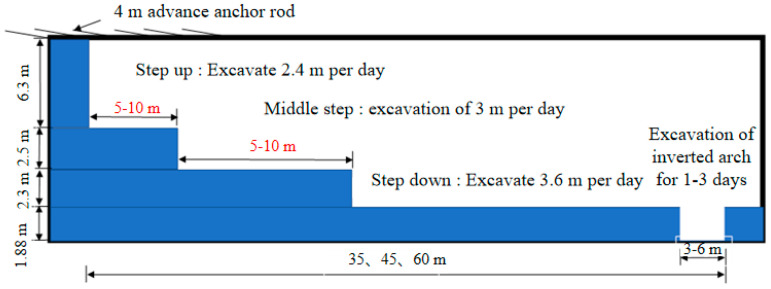
Diagram of three-step excavation.

The tri-dimensional simulation takes into account the three-step excavation technique under differing levels of surrounding rock conditions, with the excavation schematic depicted in Figure 12.

To perform the computation, the model adhered to these steps: (1) preliminary support was provided by enhancing the mechanical strength parameters of the rock and soil within the advance support zone; (2) excavation of the top steps was carried out; (3) initial support was given to the upper steps; (4) excavation of 10 steps (24.0 m) on the upper bench was done, followed by the middle bench excavation; (5) primary support was provided to the middle step; (6) construction of two excavation steps (7.2 m) for the middle step was conducted, followed by the lower step excavation; (7) initial support was offered to the lower steps; (8) excavation of the inverted arch was done after three excavation steps (10.8 m) of the lower step without initial support. The elastic-plastic body underwent an excavation cycle of 1000 steps for each step, and initial support was provided after the cycle’s completion, followed by another cycle of 1000 steps. The creep model calculation had an excavation cycle duration of 36,000 s (10 h) for each step. After the cycle’s completion, initial support was given, followed by a cycle of 1000 steps. The excavation period was 17 days for a 30 m closure distance, 22 days for a 40 m closure distance, 25 days for a 45 m closure distance, 29 days for a 50 m closure distance, 35 days for a 55 m closure distance, and 38 days for a 60 m closure distance.

### 4.4. Material Parameter Determination

The simulation of the tunnel excavation process primarily involves the fundamental physical parameters of the surrounding rock and initial and secondary support materials. The physical and mechanical parameters identified through the experiment are displayed in Table 5.

### 4.5. Creep Constitutive Law and Its Parameters

**Figure 13:**
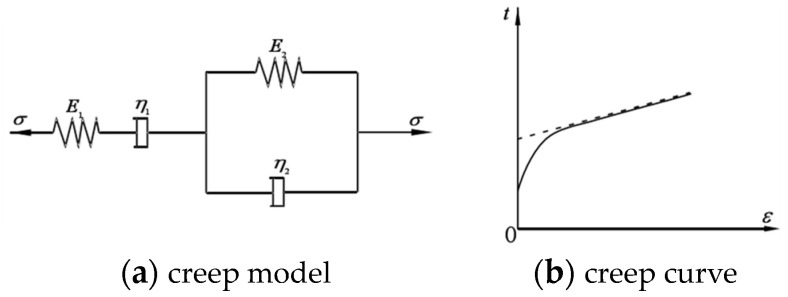
Typical burgers creep model and characteristic curve. (**a**) creep model; (**b**) creep curve.

The traditional Burgers creep model is often utilized to represent the creep curve preceding the third phase. However, it only accounts for the viscoelasticity of the rock, failing to accurately capture the creep characteristics of soft rocks, which generally demonstrate immediate plasticity, elasticity, viscoplasticity, and viscoelasticity. To rectify this, we suggest an enhanced Burgers creep model that integrates the Mohr–Coulomb criterion with the conventional Burgers creep adjustment model. Through the use of creep tests, we create a novel creep model that mimics the viscoelastic plastic traits of various rock specimens, enabling us to more precisely identify the creep characteristic parameters for each specimen. The traditional Burgers creep model, which consists of the Kelvin model and the Maxwell model in a sequential arrangement, is illustrated in Figure 13.

In the figure, *E*_M_ and *η*_M_ are the elastic modulus and viscosity coefficient of Maxwell model respectively. *E*_K_ and *η*_K_ are the elastic modulus and viscosity coefficient of the Kelvin model respectively.

Under constant axial stress σ_0_, the axial strain *ε*(*t*) is as follows:

(4) $$\varepsilon \left(t\right)=\frac{\sigma_{0}}{9K}+\frac{\sigma_{0}}{3G_{1}}+\frac{\sigma_{0}}{3G_{2}}+\frac{\sigma_{0}}{3G_{2}}e^{-E_{2}/\eta_{2}t}+\frac{\sigma_{0}}{3\eta_{1}}t$$

Within Equation (2), *K* stands for the rock specimen’s bulk modulus, *G*_1_ signifies the model’s elastic shear modulus, and *G*_2_ denotes the modulus controlling delay elasticity. *η*_1_ and *η*_2_, respectively, govern the rate of viscous flow and delay elasticity in the model.

The use of the relevant rheological experimental results in this article and the newly constructed Cvisc model can more reasonably simulate the excavation process of tunnels. We will import the relevant constitutive models into numerical calculation software through secondary development and apply them for simulation calculations.

### 4.6. Numerical Simulation-Based Analysis of Arch Settlement of V-Class Surrounding Rock Tunnel

**Figure 14:**
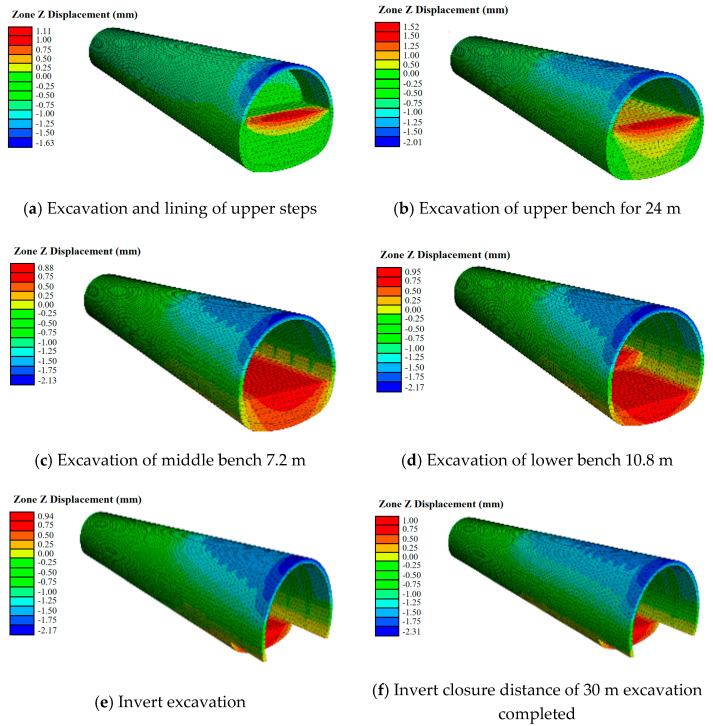
Deformation nephogram of three-step excavation tunnel with modified Burgers creep model.

The Cvisc model was utilized to perform numerical computations on the excavation model of a V-level surrounding rock three-tier tunnel. This model featured a 30 m closure distance for the inverted arch and a single excavation of 6 m for the same. The outcomes of the three-dimensional simulation are exhibited in Figure 14.

The outcomes of the numerical analysis underscore that neglecting the impact of stress release and timeliness on the mechanical and deformation characteristics of adjacent rocks in the Mohr–Coulomb constitutive model leads to a minimization of settlement and deformation at the vault during excavation. Conversely, the Cvisc constitutive model takes into consideration the strength decrease brought about by stress release and the timeliness of construction stages, providing a more accurate representation of the excavation process.

In the excavation simulation using the Cvisc model, the deformation at the arch waist of the open section continues to grow with each excavation step. In contrast, when using the Mohr–Coulomb model, the deformation at the arch waist of the open section of the inverted arch stays constant, a result that deviates notably from the actual monitored conditions. These observations emphasize that in tunnel excavation simulations, the reduction in rock strength following excavation due to stress release and the temporal aspect of excavation steps must be taken into account.

## 5. Discussion

### 5.1. The Influence of Whether Considering the Rheological Effects on Surrounding Rock

**Figure 15:**
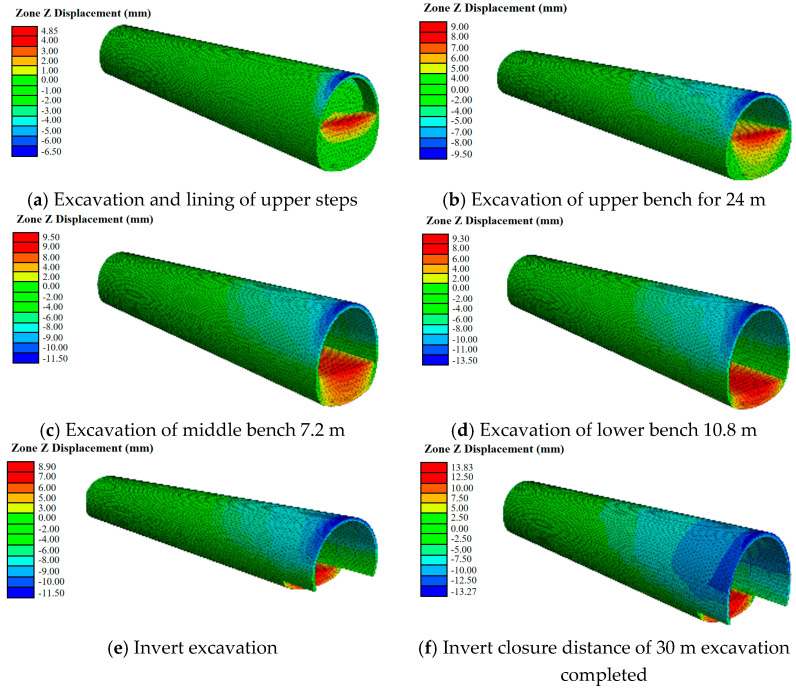
Deformation nephogram of three-step excavation tunnel with Mohr–Coulomb criterion.

Computational simulations were carried out on a V-shaped three-tier tunnel excavation model for the surrounding rock, employing the optimal elastic-plastic model grounded on the Mohr–Coulomb criterion. The inverted arch possessed a closure distance of 30 m, and a single excavation of 6 m was executed for the inverted arch. The outcomes of the three-dimensional simulation can be viewed in Figure 15.

**Figure 16:**
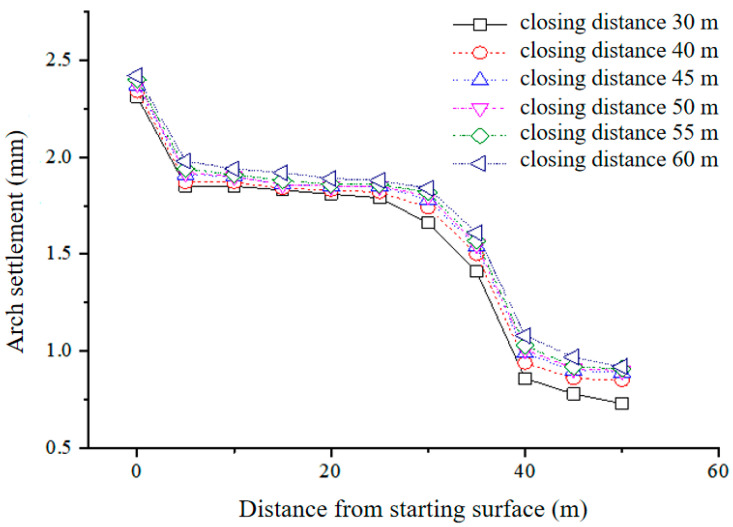
Settlement curve with Mohr–Coulomb model.

The simulation was performed for tunnel diggings with the inverted arch’s closure distances set at 30 m, 40 m, 45 m, 50 m, 55 m, and 60 m. The peak settlement curve of the vault is depicted in Figure 16, showcasing a maximum settlement deformation of a mere 2.5 mm. Nevertheless, there is no discernible relationship between settlement and the factors of time and space, which represents a slight deviation from the real-world scenario. The Cvisc model obtained from the experiment can better reflect the real situation.

### 5.2. The Influence of Tunnel Vault Settlement

(1) Impact of invert step distance on vault settlement in V-grade surrounding rock tunnel.

Utilizing the aforementioned method, we computed the settlement of the vault resulting from varying closure distances with the Burgers creep model for V-grade surrounding rock. The outcomes are depicted in Table 6. The examination indicates that when the closure distance extends from 30 m to 60 m, there is an increase in the maximum displacement of the vault from 13.33 mm to 25.1 mm.

(2) Influence of excavation length of the inverted arch of V-grade surrounding rock tunnel on vault settlement.

**Figure 17:**
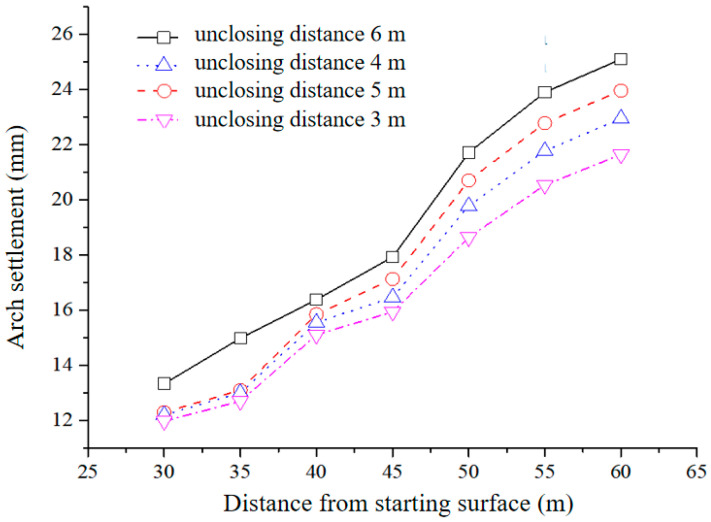
Variation curve of vault settlement.

The Burgers creep model was employed to calculate the settlement of the vault, which was caused by different lengths of the exposed inverted arch (5 m, 4 m, and 3 m) within V-grade surrounding rock. The resultant data are illustrated in Figure 17 and Table 6.

The results from the previous computations in Table 7 demonstrate that a reduction in the exposed distance of the inverted arch corresponds to a decrease in the maximum settlement of the vault. This suggests that a shorter exposed distance of the inverted arch contributes to enhanced initial support stability and a more noticeable initial support force ring effect. However, the settlement curve reveals that the maximum settlement of the vault is not particularly sensitive to the exposed distance of the inverted arch, implying that the impact of the exposed distance of the inverted arch on stability is not substantial.

(3) Influence of surrounding rock grade on vault settlement

The stability of a tunnel is largely determined by the characteristics of the rocks that surround it. Thus, to explore the correlation between the closure distance of the inverted arch and the maximum settlement of the initial support vault under different grades of the surrounding rock, the strength of the surrounding rocks was adjusted based on the model previously mentioned. The Burgers creep model parameters for various grades of surrounding rocks are detailed in Table 6.

**Figure 18:**
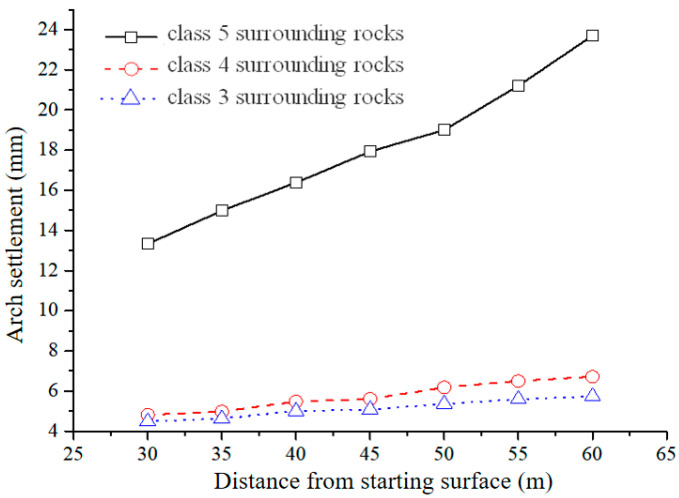
Tunnel vault settlement curves caused by different levels of surrounding rock and different excavation methods.

The findings derived from the Burgers creep model are illustrated in Figure 18 and Table 8. It’s noticeable that the maximum settlement value of a vault in V-grade surrounding rock exceeds that of III- and IV-grade surrounding rocks, and the maximum settlement of a vault in IV-grade surrounding rock is marginally greater than that of III-grade surrounding rock vault across each closure distance. Moreover, as the closure distance expands, the maximum settlement value of the vault escalates as well.

The information in Table 6 indicates that a rise in the grade of the surrounding rocks leads to a substantial decrease in the displacement value of the tunnel vault, suggesting a high sensitivity of the vault settlement to alterations in the grade of surrounding rocks. These findings imply that the stability of the initial support is intimately associated with the grade of the surrounding rocks. More specifically, when the conditions of the surrounding rocks are favorable, the closure distance of the inverted arch can be suitably extended, and the exposed distance of the inverted arch can be increased to accommodate the operation of large machinery. On the other hand, when the conditions of the surrounding rocks are unfavorable, the closure distance of the inverted arch should be reduced, and the exposed distance of the inverted arch should be minimized, while the initial support ought to be applied swiftly to preserve the stability of the surrounding rock. Furthermore, extending the length of circular footage and diminishing the time of circular footage under favorable surrounding rock conditions can notably boost the stability of both the surrounding rock and the tunnel.

### 5.3. Reasons for Using the Modified Model Proposed in This Paper and Comparison

In discussions about tunnel settlement with distance from the starting face, many experts have employed different constitutive modeling approaches. Initially, numerous experts used elastic-plastic constitutive models for simulation and analysis, such as the Mohr–Coulomb model [34,35,36,37] and the Druck–Prager model [38]. These methods have significant drawbacks: they primarily describe the short-term elastic and plastic deformations of the rock mass and are insufficient for capturing time-dependent deformations. Consequently, they may fail to accurately predict long-term deformations, overlook the viscous behavior of rocks, and inadequately describe the actual deformation process of surrounding rock. These shortcomings are particularly pronounced in complex rock rheological characteristics and can lead to substantial calculation deviations in step distance control, potentially resulting in serious safety incidents. On the other hand, creep constitutive models better reflect the time-dependent deformation behavior of rocks and describe long-term rock stress-strain relationships. They align more closely with the mechanical properties of surrounding rock and typically integrate elastic, viscous, and plastic characteristics, providing a more comprehensive description of the deformation process under real conditions.

For predicting rock deformation, commonly used creep constitutive models include the traditional Burgers creep model and the Kelvin creep model [39,40]. However, these models often only account for the viscoelasticity of the rock, failing to accurately capture the creep characteristics of soft rocks, which generally exhibit immediate plasticity, elasticity, viscoplasticity, and viscoelasticity. To address this, we suggest an enhanced Burgers creep model that integrates the Mohr–Coulomb criterion with the conventional Burgers creep adjustment model. This improved model, with its comprehensive consideration of viscous, elastic, and plastic rheological characteristics, generally performs better than the Kelvin and Burgers models in complex rheological behaviors and variable engineering conditions, especially for long-term deformation predictions.

**Figure 19:**
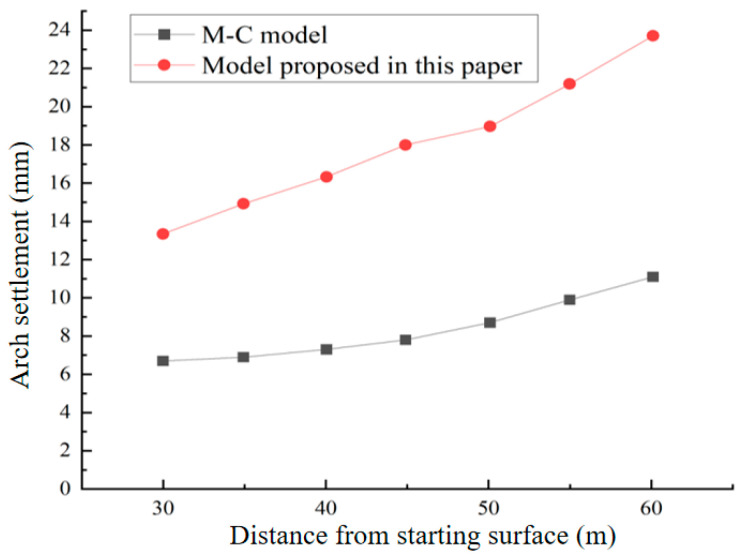
Results comparison of the model proposed in this paper and the M-C model.

Figure 19 shows the simulation results for Class 5 surrounding rock using both the creep model proposed in this study and the Mohr–Coulomb model. From Figure 18, it is evident that the Mohr–Coulomb model, which does not consider the time effects of tunnel construction on soil deformation, primarily reflects immediate stress redistribution. Consequently, the displacement and stress calculation results are underestimated, making it potentially dangerous for design and construction. In contrast, the improved model proposed in this paper, which accounts for the time effects of tunnel construction, provides results that are more conducive to ensuring the safety of construction design and better align with the mechanical properties of the surrounding rock.

## 6. Conclusions

(1) Through the rheological test research on siltstone and sandstone, it was found that the rheological properties of siltstone are more in line with the Burgers model, while the rheological properties of sandstone are more in line with the Cvisc model.

(2) The deformation mechanism of the Burgers time effect in siltstone surrounding rock rheology was revealed, and corresponding values of model parameters were obtained through fitting. In low-stress conditions, as the stress level increases, the shear modulus of the Maxwell model decreases while the viscosity coefficient increases. The shear modulus and viscosity coefficient of the Kelvin model both increase with increasing load. Furthermore, as the surrounding rock conditions improve, the values of these parameters increase.

(3) The deformation mechanism of the Cvisc time effect in sandstone surrounding rock rheology was revealed, and corresponding values of model parameters were obtained through fitting. The elastic modulus of the Maxwell model increases with increasing load, while the viscosity coefficient of the surrounding rock decreases. The variations of the Kelvin creep parameters are not significant. However, under different surrounding rock conditions, all parameters consistently increase to some extent as the surrounding rock grade decreases.

(4) A reduction in the closure distance of the inverted arch leads to a decrease in the maximum settlement of the vault. This implies that the stability of the initial support is enhanced when the closure distance is smaller and the impact of the initial support force ring is more noticeable. The maximum settlement of the vault isn’t sensitive to the exposed distance of the inverted arch. This suggests that the distance of the inverted arch doesn’t significantly affect the stability of the initial support.

(5) After the mechanized process, the sealing distance of the inverted arch in V-grade surrounding rock can be maintained within a 55 m range, while in IV-grade surrounding rock, it can be kept within a 70 m range. For an inverted arch in III-grade surrounding rock, the sealing distance can be adjusted according to the specific circumstances. The excavation length of the inverted arch in V-grade surrounding rock can be kept within a 5 m range at a time.

(6) The step method is appropriate for excavation in Grade III, IV, and V surrounding rocks, and the length can be adjusted based on the settlement of the vault resulting from the excavation.

## Figures and Tables

**Table 1:** Specimens situation table.

Group Number	Average Diameter/mm	Average Height/mm	Surrounding Rock Grade
1	50.11	100.03	III
2	49.93	99.88	IV
3	50.04	99.93	V

**Table 2:** The physical and mechanical parameters of test specimens.

Group Number	*ρ*/(g/cm^3^)	*E_S_*/(GPa)	*υ*	*c*/(kPa)	*φ*/(°)	Surrounding Rock Grade
1	2.32	11.8	0.18	120.32	28.65	III
2	2.39	12.8	0.21	125.45	25.43	IV
3	2.51	12.3	0.19	135.32	30.54	V

Note: The values in the table are the averages of each group of data.

**Table 3:** Stress level of sandstone under triaxial compression creep loading.

Group Number	Level 1 MPa	Level 2 MPa	Level 3 MPa	Level 4 MPa	Level 5 MPa	Level 6 MPa	Level 7 MPa	Level 8 MPa	Level 9 MPa	Level 10 MPa
1	36.2	43.6	51.2	59.1	66.3	74.5	82.2	90.3	97.3	105.2
2	30.1	38.5	46.2	54.1	62.2	69.5	77.2	85.2	92.3	/
3	26.3	33.5	41.2	49.1	57.4	64.5	/	/	/	/

**Table 4:** Tunnel arch settlement monitor.

Step	Left Vault Settling/mm	Right Vault Settling/mm	Step	Left Vault Settling/mm	Right Vault Settling/mm
1	0.0	0.0	22	0.7	8.4
2	0.0	0.0	23	1.1	8.7
3	0.0	0.0	24	1.6	8.9
4	0.0	0.0	25	2.1	9.1
5	0.0	0.1	26	2.8	9.2
6	0.0	0.1	27	3.5	9.4
7	0.0	0.2	28	4.2	9.5
8	0.0	0.3	29	4.9	9.5
9	0.0	0.5	30	5.6	9.6
10	0.0	0.7	31	6.2	9.7
11	0.0	1.1	32	6.6	9.8
12	0.0	1.6	33	6.9	9.8
13	0.0	2.3	34	7.1	9.9
14	0.0	2.9	35	7.2	10.0
15	0.0	3.7	36	7.4	10.0
16	0.0	4.5	37	7.5	10.1
17	0.1	5.3	38	7.6	10.1
18	0.1	6.1	39	7.7	10.2
19	0.1	6.8	40	7.8	10.3
20	0.2	7.5	41	7.9	10.3
21	0.4	8.0	42	8.0	10.4

**Table 5:** Basic parameters of simulated materials.

Material Science	Deformation Modulus/(GPa)	Poisson’s Ratio	Cohesion/(MPa)	Internal Friction Angle/(°)	Density/(kg/m^3^)
V-grade surrounding rock	18	0.30	0.05	25	2000
Initial support	1.6	0.4	/	/	2500
Secondary support	20	0.25	/	/	2500
IV-grade surrounding rock	30	0.25	0.35	30	2200
III-grade surrounding rock	40	0.22	0.50	35	2500

**Table 6:** Maximum displacement of a vault under different closure distances of an inverted arch in V-grade surrounding rock.

Closing Distance (m)	Excavation Time (Day)	Maximum Displacement of Vault (mm)
30	17	13.33
40	22	16.38
45	25	17.93
50	29	21.71
55	35	23.9
60	38	25.1

**Table 7:** Maximum displacement of inverted arch without closure of 3–6 m in V-grade rock mass.

Closing Distance (m)	Unenclosed Inverted Arch 6 m Maximum Displacement of Vault (mm)	Unenclosed Inverted Arch 5 m Maximum Displacement of Vault (mm)	Unenclosed Inverted Arch 4 m Maximum Displacement of Vault (mm)	Unenclosed Inverted Arch 3 m Maximum Displacement of Vault (mm)
30	13.33	12.3	12.2	11.98
35	14.98	13.1	13	12.7
40	16.38	15.85	15.55	15.1
45	17.93	17.13	16.47	15.94
50	21.71	20.7	19.78	18.64
55	23.9	22.78	21.77	20.54
60	25.1	23.96	22.95	21.64

**Table 8:** Numerical results.

Closing Distance (m)	Maximum Settlement of Class V Surrounding Rock Arch (mm)	Maximum Settlement of Class IV Surrounding Rock Arch (mm)	Maximum Settlement of Class III Surrounding Rock Arch (mm)
30	13.33	4.82	4.49
35	14.98	4.98	4.63
40	16.38	5.48	5.00
45	17.93	5.61	5.08
50	21.71	6.18	5.36
55	23.9	6.5	5.6
60	25.1	6.72	5.74

## Data Availability

Data is contained within the article.
